# Comparison of immunological characteristics between paired mismatch repair-proficient and -deficient colorectal cancer patients

**DOI:** 10.1186/s12967-018-1570-z

**Published:** 2018-07-13

**Authors:** Shou-Sheng Liu, Yuan-Zhong Yang, Chang Jiang, Qi Quan, Qian-Kun Xie, Xiao-Pai Wang, Wen-Zhuo He, Yu-Ming Rong, Ping Chen, Qiong Yang, Lin Yang, Bei Zhang, Xiao-Jun Xia, Peng-Fei Kong, Liang-Ping Xia

**Affiliations:** 1 State Key Laboratory of Oncology in South China, Collaborative Innovation Centre for Cancer Medicine, Sun Yat-sen University Cancer Center, No. 651 Dongfeng East Road, Guangzhou, 510060 People’s Republic of China; 2 Department of the VIP Region, Sun Yat-sen University Cancer Center, Guangzhou, 510060 People’s Republic of China; 3 Department of Pathology, Sun Yat-sen University Cancer Center, Guangzhou, 510060 People’s Republic of China; 4 Department of Pathology, Guangzhou First People’s Hospital, Guangzhou Medical University, Guangzhou, 510080 People’s Republic of China; 5 Department of Oncology, Sun Yat-Sen Memorial Hospital, Guangzhou, 510000 People’s Republic of China

**Keywords:** Colorectal cancer, Mismatch repair-deficient, Mismatch repair-proficient, MHC class I molecules, Programmed death-1

## Abstract

**Background:**

Currently, mismatch repair-deficient (dMMR) status is a promising candidate for targeted immune checkpoint inhibition therapy in colorectal cancer (CRC) patients, however, the potential immunological mechanism has not yet been well clarified and some other predictors need to be excavated as well.

**Methods:**

We collected 330 CRC patients by the match of mismatch repair-proficient (167) and dMMR (163), explored the relationship between MMR status and some important immune molecules including MHC class I, CD3, CD4, CD8, CD56, programmed death-1 and programmed death ligand-1, and investigated the risk factors for dMMR status as well as low MHC class I expression. The Pearson Chi square test was used for analyzing the associations between clinicopathological and immune characteristics and MMR status, and two categories logistic regression model was used for univariate and multivariate analysis to predict the odds ratio of risk factors for dMMR status and low MHC class I expression.

**Results:**

Multivariate logistic regression analysis showed that low MHC class I and CD4 expression and high CD8 expression were significant risk factors for dMMR status [odds ratio (OR) = 24.66, 2.94 and 2.97, respectively; all *p* < 0.05] and dMMR status was the only risk factor for low MHC class I expression (OR = 15.34; *p* < 0.001).

**Conclusions:**

High CD8 and low MHC class I expression suggests the contradiction and complexity of immune microenvironment in dMMR CRC patients. Some other immunocytes such as CD56^+^ cells might also participate in the process of immune checkpoint inhibition, whereas needs further investigations.

## Background

In the past few years, immune checkpoint inhibitors including anti-programmed death-1 (PD-1), programmed death ligand-1 (PD-L1) and CTL-associated antigen-4 (CTLA-4) have yielded positive effect in a series of malignancies such as melanoma, non-small cell lung cancer, renal cell carcinoma, bladder cancer, and Hodgkin’s lymphoma [1–3]. Nevertheless, anti-PD-1 therapy has not shown encouraging outcomes in colorectal cancer (CRC) until 2015, when a phase II study showed that 40% of mismatch repair-deficient (dMMR) CRC patients achieved an objective response, while none of the patients with mismatch repair-proficient (pMMR) status responded to anti-PD-1 therapy [4].

Currently, dMMR CRC patients are suggested to be promising candidates for targeted immune checkpoint inhibition therapy. The most mentioned explanation for the enhanced activity of anti-PD-1 therapy in dMMR patients is the generation of neo-antigens, which then activate T cell response to tumor cells [4, 5]. Previous studies have also presented some other possible mechanisms such as changes in signaling pathways that lead to more cytokine and chemokine expression [6–8]. However, which immune cells and how they participate in the process remains unclear. Since approximately only 10–20% of all colorectal tumors (about 20% in stage II, 12% in stage III and 4% in stage IV) have microsatellite instability (MSI) or to say dMMR [9–12], it is not easy to obtain enough dMMR cases to investigate the concrete mechanisms. Besides, as only less than half dMMR CRC patients benefit from anti-PD-1 therapy, MMR status might not be the only positive predictor for anti-PD-1 therapy, some other predictive indexes should be explored to combine with MMR status in order to better predict the curative effect.

In this study, we enrolled 330 CRC patients by the match of pMMR (167) and dMMR (163) and explored the relationship between MMR status and other clinicopathologic characteristics, especially some important anti-tumor immune molecules such as MHC class I, CD3, CD4, CD8, CD56, PD-1 and PD-L1 to explain the better effect of anti-PD-1 therapy in dMMR group and explore some other possible efficacy predictors besides MMR status.

## Methods

### Patients and specimens

We collected surgical specimens from 330 patients who underwent primary surgery and diagnosed with stage I to IV CRC according to the seventh edition of the TNM Classification from March 2009 to December 2013 at Sun Yat-Sen University Cancer Center in Guangzhou, China. Clinicopathological characteristics are summarized in Table 1. The obtained surgical specimens were fixed in 10% formalin and embedded in paraffin for further use.

**Table 1:** Clinicopathological and immune characteristics of CRC patients based on MMR status

Characteristics	pMMR	dMMR	r	*p* value
Number of cases (n, %)				
Age at diagnosis (years)			0.194	*< 0.001*
< 60	80 (47.9)	110 (67.5)		
≥ 60	87 (52.1)	53 (32.5)		
Gender				0.24
Male	96 (57.5)	104 (63.8)		
Female	71 (42.5)	59 (36.2)		
Primary tumor site			0.264	*< 0.001*
Left colon	81 (48.5)	41 (25.2)		
Right colon	47 (28.1)	86 (52.8)		
Rectum	39 (23.4)	36 (22.1)		
Stage (7th AJCC)				0.965
I	27 (16.2)	26 (16.0)		
II	89 (53.3)	85 (52.1)		
III	42 (25.1)	41 (25.2)		
IV	9 (5.4)	11 (6.7)		
Tumor stage			0.193	*0.012*
T1	8 (4.8)	5 (3.1)		
T2	25 (15.0)	24 (14.7)		
T3	118 (70.7)	95 (58.3)		
T4a	11 (6.6)	27 (16.6)		
T4b	5 (3.0)	12 (7.4)		
Node stage				0.819
Negative	119 (71.3)	118 (72.4)		
Positive	48 (28.7)	45 (27.6)		
Negative lymph nodes			0.179	*0.001*
Low (< 14)	102 (61.1)	70 (42.9)		
High (≥ 14)	65 (38.9)	93 (57.1)		
Tumor histological grade			0.212	*0.001*
Well-differentiated	2 (1.2)	1 (0.6)		
Moderately differentiated	109 (65.3)	77 (47.2)		
Poorly differentiated	27 (16.2)	27 (16.6)		
Mucinous	29 (17.4)	58 (35.6)		
Nerve invasion			0.12	*0.029*
No	142 (85.0)	151 (92.6)		
Yes	25 (15.0)	12 (7.4)		
Vascular invasion			0.123	*0.025*
No	143 (85.6)	152 (93.3)		
Yes	24 (14.4)	11 (6.7)		
MHC class I expression			0.454	*< 0.001*
Low	12 (7.2)	88 (54.0)		
High	155 (92.8)	75 (46.0)		
CD3 expression			0.026	0.661
Low	66 (43.4)	63 (46.0)		
High	86 (56.6)	74 (54.0)		
CD8 expression			0.156	*0.009*
Low	83 (59.3)	57 (43.5)		
High	57 (40.7)	74 (56.5)		
CD56 expression			0.228	*< 0.001*
Low	41 (24.7)	75 (47.2)		
High	125 (75.3)	84 (52.8)		
CD4 expression			0.2	*< 0.001*
Low	49 (30.1)	78 (50.0)		
High	114 (69.9)	78 (50.0)		
PD-1 expression			0.043	0.483
Low	62 (46.3)	55 (42.0)		
High	72 (53.7)	76 (58.0)		
PD-L1 expression			0.133	*0.015*
Negative	77 (46.1)	97 (59.5)		
Positive	90 (53.9)	66 (40.5)		

r: contingency coefficient

### Immunohistochemistry

To detect MMR proteins, mouse anti-MLH1, MSH2, MSH6 and PMS2 monoclonal antibodies (DAKO, Denmark) were used. Mouse monoclonal antibodies against pan MHC class I molecule (EMR8-5, Hokudo, Japan), CD3, CD4, CD8 and CD56 (DAKO, Denmark) as well as rabbit monoclonal antibodies against PD-1 (Abcam, UK) and PD-L1 (Cell Signaling Technology, USA) were used to test the expression of corresponding proteins. Tissue sections of 4 μm thickness were deparaffinized in xylene, washed and dehydrated with graded ethanol, and then dipped in methanol with 0.3% hydrogen peroxide for more than 15 min followed by antigen retrieval. The slides were then incubated with the primary antibody at 4 °C overnight. After been washed three times with phosphate-buffered saline (PBS), the slides were subsequently co-incubated with the biotin-linked secondary antibodies according to the manufacturer’s instructions. Slides were then counter-stained with haematoxylin for color reaction, washed and dehydrated in graded ethanol, ultimately mounted under a cover slip. Each slide was examined by three pathologists to obtain coincident immunohistochemical (IHC) results.

### Scoring of immunohistochemistry

Both intratumoral and interstitial areas were included for analyzing the presence of CD3, CD4^+^, CD8^+^ and CD56^+^ cells using a modified semi-quantitative scoring method: 0 = “none” (no stained cells), 1 = “focal” (< 10% stained cells), 2 = “moderate” (10–40% stained cells), and 3 = “severe” (> 40% stained cells) [13, 14]. Cases of scores ≤ 1 were divided into low-expression group and scores ≥ 2 were considered to be high-expression group. The expression of MHC class I on tumor cells was graded as previously described: score 1 (< 20% positive cells), score 2 (20–80% positive cells), and score 3 (> 80% positive cells). Populations of scores 1 and 2 were classified into low-expression group and score 3 was regarded as high-expression group [15, 16]. PD-1 expression on lymphocytes was evaluated using a semi-quantitative scoring system (0 = none, 1 = < 5% of lymphocytes, 2 = 5–50% of lymphocytes, 3 ≥ 50% of lymphocytes). Scores 0 and 1 were graded as low and scores 2 and 3 were graded as high [13, 17]. PD-L1 expression on the surface of tumor cells and tumor-infiltrating immune cells was divided into two groups: specimens with < 5% membranous expression indicated “negative”, while ≥ 5% was considered “positive” [1]. Tumors lacking any one of MLH1, MSH2, PMS2, or MSH6 expression were identified as dMMR, only if tumors that express of all of the four markers were considered pMMR.

### Statistical analysis

Statistical analyses were conducted using SPSS 22.0 software (SPSS Inc, USA). The Pearson Chi square test was used to assess the association between categorical variables. Two categories logistic regression model was used for univariate and multivariate analysis to predict the odds ratio (OR) of individual factors for dMMR status and low MHC class I expression. A *p* value of less than 0.05 was considered statistically significant.

## Results

### Patient characteristics

We selected the patients by the match of pMMR (167) and dMMR (163), including 255 colon cancer (left: 122; right: 133) and 75 rectum cancer patients. The clinicopathological characteristics of the 330 cases are listed in Table 1. Median age at the time of diagnosis was 57 years range from 22 to 84, and 60.6% were male patients. Majority of the patients were in stage II (52.7%) and III (25.2%), especially in T3 (64.5%) and N0 stage (71.8%). Moderately differentiated adenocarcinoma accounted for 56.4%, while well-differentiated adenocarcinoma made up only 0.9% of all pathological types. A total of 37 (11.2%) and 35 (10.6%) patients appeared nerve and vascular invasion, respectively. As negative lymph node represented the immune status against cancer, we incorporated this index into the study, and found no obvious difference between low (< 14; 52.1%) and high (≥ 14; 47.9%) group, according to the median of negative lymph nodes.

### Relationship between MMR status and clinicopathologic and immunological features

**Fig. 1:**
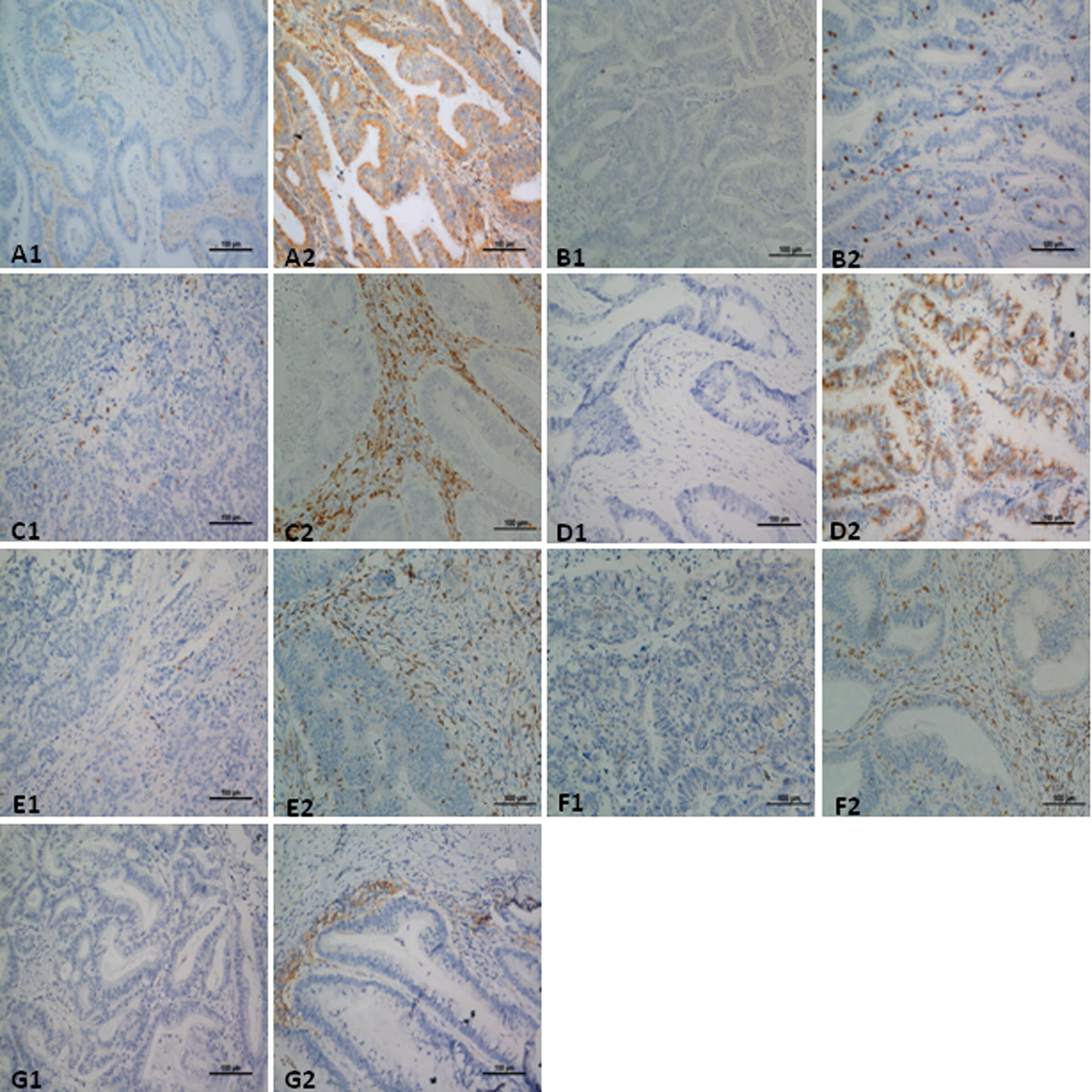
Representative IHC staining of immune molecules in CRC tissues. MHC class I expressed on tumor cells; CD3, CD4, CD8 and CD56 expressed in both intratumoral and interstitial areas; PD-1 expressed on lymphocytes; and PD-L1 expressed on tumor cells and tumor-infiltrating immune cells were evaluated. Low and high expression of MHC class I (**A1** score 1, **A2** score 3), CD3 (**B1** score 1, **B2** score 2), CD4 (**C1** score 1, **C2** score 3), CD8 (**D1** score 0, **D2** score 3), CD56 (**E1** score 1, **E2** score 3) and PD-1 (**F1** score 0, **F2** score 3); Negative and positive expression of PD-L1 (**G1**, **G2**). Images represent 200× microscopic fields used for analysis

Aberrant protein expression in dMMR patients was listed in Table 2. Single negative expression rates of MLH1, MSH2, MSH6 and PMS2 were 6.7, 3.1, 21.5 and 5.5%, respectively. Concurrent negative expression of two, three and all the four MMR proteins were observed in 52.1, 8.0 and 3.1% dMMR patients, respectively. We investigated whether MMR status has relationship with other clinical features, especially immune molecules. Representative IHC stainings for immune molecules were displayed in Fig. 1. As shown in Table 1, the MMR status was significantly correlated with the expression of MHC class I and CD8, and dMMR group displayed much less MHC class I and higher CD8 expression than pMMR group (all *p* < 0.01). Besides, CD56^+^ cell, CD4^+^ cell and PD-L1 expression were also reduced in dMMR group (all *p* < 0.05). Other characteristics such as age, primary tumor site, tumor stage, histological grade, number of negative lymph nodes, nerve and vascular invasion were also significantly correlated with the status of MMR (all *p* < 0.05). Univariate logistic regression analysis confirmed the above findings, however, when the above meaningful factors entered into multivariate logistic regression model (Table 3), negative lymph nodes, tumor histological grade, nerve invasion, CD56 and PD-L1 expression were found not to be significant risk factors for dMMR status (all *p* > 0.05).

**Table 2:** Aberrant protein expression in dMMR patients

Aberrant protein expression	Number of cases (n, %)
MMR (1)	60 (36.8)
MLH1	11 (6.7)
MSH2	5 (3.1)
MSH6	35 (21.5)
PMS2	9 (5.5)
MMR (2)	85 (52.1)
MLH1 + MSH2	1 (0.6)
MLH1 + MSH6	2 (1.2)
MLH1 + PMS2	49 (30.1)
MSH2 + MSH6	30 (18.4)
MSH2 + PMS2	2 (1.2)
MSH6 + PMS2	1 (0.6)
MMR (3)	13 (8.0)
MLH1 + MSH2 + PMS2	4 (2.5)
MLH1 + MSH6 + PMS2	6 (3.7)
MLH1 + MSH2 + MSH6	2 (1.2)
MSH2 + MSH6 + PMS2	1 (0.6)
MMR (4)	5 (3.1)

MMR (1): negative expression of only one MMR protein

MMR (2): concurrent negative expression of two MMR proteins

MMR (3): concurrent negative expression of three MMR proteins

MMR (4): concurrent negative expression of four MMR proteins

**Table 3:** Logistic regression analysis of risk factors for dMMR status

Factors	Univariate analysis		Multivariate analysis	
	OR (95% CI)	*p*-value	OR (95% CI)	*p*-value
Age at diagnosis (≥ 60)	0.44 (0.28–0.69)	< 0.001	0.48 (0.25–0.94)	*0.032*
Gender (female)	0.77 (0.49–1.19)	0.241		
Primary tumor site		< 0.001		*0.001*
Left colon	0.55 (0.30–0.99)	0.045	0.43 (0.17–1.09)	0.076
Right colon	1.98 (1.11–3.53)	0.02	2.28 (0.86–6.07)	0.098
Rectum (*reference*)	–	–	–	–
Stage (7th AJCC)		0.965		
I	0.79 (0.28–2.21)	0.651		
II	0.78 (0.31–1.98)	0.603		
III	0.80 (0.30–2.13)	0.653		
IV (*reference*)	–	–		
Tumor stage		0.007		*0.004*
T1	0.26 (0.07–0.90)	0.034	0.04 (0.01–0.33)	0.002
T2	0.39 (0.18–0.88)	0.024	0.38 (0.11–1.37)	0.139
T3	0.33 (0.17–0.63)	0.001	0.20 (0.07–0.59)	0.004
T4 (*reference*)	–	–	–	–
Node stage (positive)		0.819		
Negative lymph nodes (high)	2.09 (1.34–3.24)	0.001	1.10 (0.52–2.30)	0.810
Tumor histological grade		0.002		0.588
Well-differentiated	0.25 (0.02–2.87)	0.266	7.91 (0.38–163.50)	0.181
Moderately differentiated	0.35 (0.21–0.60)	< 0.001	1.12 (0.48–2.63)	0.788
Poorly differentiated	0.50 (0.25–1.00)	0.051	0.93 (0.30–2.89)	0.896
Mucinous (*reference*)	–		–	–
Nerve invasion (no)	2.22 (1.07–4.58)	0.032	1.80 (0.59–5.45)	0.299
Vascular invasion (no)	2.32 (1.10–4.91)	0.028	3.66 (1.04–12.98)	*0.044*
MHC class I expression (low)	15.16 (7.81–29.42)	< 0.001	24.66 (8.74–69.64)	*<* *0.001*
CD3 expression (high)	0.90 (0.57–1.43)	0.662		
CD4 expression (high)	0.43 (0.27–0.68)	< 0.001	0.34 (0.14–0.83)	*0.017*
CD8 expression (high)	1.89 (1.17–3.06)	0.01	2.97 (1.30–6.78)	*0.01*
CD56 expression (high)	0.37 (0.23–0.59)	< 0.001	0.92 (0.78–1.08)	0.294
PD-1 expression (high)	1.19 (0.73–1.93)	0.483		
PD-L1 expression (positive)	0.58 (0.38–0.90)	0.015	0.55 (0.27–1.13)	0.103

OR: odds ratio; 95% CI: 95% confidence interval

Only the meaningful factors (*p *< 0.05) in univariate analysis were brought into the multivariate analysis

### Risk factor analysis for low MHC class I expression

As dMMR status significantly correlated with low MHC class I expression, and MHC class I molecules play an important role in anti-tumor process, we next carried out the univariate and multivariate logistic regression analysis to explore the risk factors for low MHC class I expression. As shown in Table 4, age under 60, dMMR status, and low CD4 and CD56 expression were risk factors for low MHC class I expression in univariate model (all *p* < 0.05). Besides, tumor stage and tumor histological grade were also meaningful variables in the model (*p* < 0.05). However, when it came to multivariate logistic regression model, dMMR status was the only significant risk factor for low MHC class I expression (OR = 15.34, 95% confidence interval (CI) 7.24–32.50, *p* < 0.001).

**Table 4:** Logistic regression analysis of risk factors for low MHC class I expression

Factors	Univariate analysis		Multivariate analysis	
	OR (95% CI)	*p*-value	OR (95% CI)	*p*-value
Age at diagnosis (≥ 60)	0.60 (0.37–0.98)	0.042	0.99 (0.54–1.85)	0.998
Gender (female)	0.72 (0.44–1.17)	0.187		
Primary tumor site		0.136		
Left colon	0.79 (0.41–1.50)	0.464		
Right colon	1.36 (0.74–2.50)	0.323		
Rectum (*reference*)	–	–		
Stage (7th AJCC)		0.245		
I	0.47 (0.17–1.35)	0.161		
II	0.38 (0.15–0.97)	0.044		
III	0.43 (0.16–1.17)	0.097		
IV (*reference*)	–	–		
Tumor stage		0.005		0.122
T1	0.89 (0.27–2.99)	0.849	6.78 (0.97–47.42)	0.054
T2	0.37 (0.16–0.86)	0.02	0.91 (0.22–3.72)	0.897
T3	0.35 (0.19–0.65)	0.001	0.71 (0.33–1.52)	0.377
T4 (*reference*)	–	–	–	–
Node stage (positive)	0.92 (0.54–1.56)	0.753		
Negative lymph nodes (high)	1.42 (0.89–2.28)	0.143		
Tumor histological grade		0.004		0.117
Well-differentiated	0.00	0.999	0.00	0.999
Moderately differentiated	0.38 (0.22–0.65)	< 0.001	0.47 (0.23–0.94)	0.034
Poorly differentiated	0.76 (0.38–1.52)	0.436	0.99 (0.42–2.38)	0.989
Mucinous (*reference*)	–	–	–	–
Nerve invasion (no)	1.66 (0.73–3.77)	0.227		
Vascular invasion (no)	1.10 (0.51–2.38)	0.814		
MMR status (dMMR)	15.16 (7.81–29.42)	< 0.001	15.34 (7.24–32.50)	*< 0.001*
CD3 expression (high)	0.91 (0.54–1.52)	0.714		
CD4 expression (high)	0.57 (0.35–0.93)	0.025	1.05 (0.57–1.96)	0.871
CD8 expression (high)	1.42 (0.83–2.42)	0.198		
CD56 expression (high)	0.49 (0.30–0.79)	0.004	0.92 (0.50–1.70)	0.795
PD-1 expression (high)	0.95 (0.56–1.61)	0.855		
PD-L1 expression (positive)	0.70 (0.43–1.12)	0.133		

OR: odds ratio; 95% CI: 95% confidence interval

Only the meaningful factors (*p* < 0.05) in univariate analysis were brought into the multivariate analysis

## Discussion

Anti-PD-1 therapy has shown good therapeutic effect in dMMR rather than pMMR CRC patients; however, the underlying mechanism remains not very clear. Several studies have shown that T cell activation by large proportion of mutant neoantigens in dMMR cancers make them sensitive to immune checkpoint blockade, and that immune score based on CD3^+^ and CD8^+^ T cell counts varied between dMMR and pMMR goups and had positive prognostic significance in CRC patients [18, 19]. These researches have revealed some immunological mechanisms in the process and the immunological characteristic differences between the two groups. We designed this retrospective study to provide some possible explanations in addition to T cell participation, as well as offer some other potential indexes in combination with MMR status for the efficacy prediction of immune checkpoint inhibition therapy in CRC patients.

Previous studies have shown that loss of MHC class I expression is very common in up to 60% of dMMR CRC patients [20, 21], in accordance with these researches, our result also revealed that about 54% (88/163) of dMMR patients had low expression rate of MHC class I molecules.

As known to all, MHC class I molecule is a crucial media expressed on the surface of cancer cells for the recognition by CD8^+^ cytotoxic lymphocytes (CTL). The MMR deficiency provoked enhanced immune response might be weaken by the immune evasion mediated by loss of MHC class I [22]. How could this phenomenon be in agreement with the anti-PD-1 clinical data that only dMMR patients can benefit? Former researches provided some possible explanations: First, antigen-presenting cells such as dendritic cells may cross-present MHC class I-restricted tumor related antigens to CTL and trigger the process of tumors killing. The mechanism has been confirmed in a mouse model [23]. Second, based on the findings that CD4^+^ T cells in MSI tumor environment express higher levels of PD-1 compared with microsatellite stable (MSS) tumors and CD4^+^ T cells could recognize mutated antigens in melanoma, they propose CD4^+^ T cells might play an important role in the anti-PD-1 therapy process [24, 25]. Furthermore, MHC class II-restricted T cell may also play an important role in the process, which has been verified in a pre-clinical CRC model [26]. In addition, cellular stress caused by high level DNA damage in dMMR tumors might be sensed by and activate innate immune cells [4].

In our studies, we found that dMMR status was associated with low CD4 expression and high CD8 expression, and there was no significant correlation between MMR status and CD56. In addition, dMMR status was the only risk factor for low MHC class I expression. Previous research showed that tumors from dMMR CRC patients contained a greater density of CD8^+^ T cells and higher expression of PD-L1 than did tumors from pMMR patients in stage IV [4]. The inconsistency of PD-L1 expression between our studies is probably because our data were from stage I–IV especially stage II, and PD-L1 expression both on the surface of tumor cells and tumor-infiltrating immune cells was counted. As only 40% of dMMR CRC patients achieved an objective response to anti-PD-1 therapy, we infer that only the CRC cells of high MHC class I expression could be recognized and killed by CD8^+^ T cells, because previous studies have shown that about 60% of dMMR CRC patients lack of MHC class I expression. Our data also showed that approximate 46% of cases (75/163) in dMMR group displayed high MHC class I expression, which is close to the 40% response rate.

CD56 was used as a biomarker for NK cells existing in the colorectal tumor microenvironment in majority of IHC studies [27, 28], so we assessed for the presence of NK cell infiltration in CRC tissues using the expression of CD56. Based on our findings, and the conception that loss or alteration of MHC class I molecule is the essential condition for NK cell activation [29–31], we could speculate that CD56^+^ NK cell might also play a role in the process of immune checkpoint inhibition therapy. However, current result is insufficient to prove the above two hypotheses, and subsequent researches should be carried out to further confirm our assumptions. Some other mechanisms should also be taken into account, for example, subgroup of CD4^+^ cell and MHC class II should be detected to evaluate the role of helper T cells and MHC class II molecule in the process. Moreover, it would be even better to compare the immune molecules, especially MHC class I molecules before and after anti-PD-1 therapy. If MHC class I molecule were up-regulated, the doubts would be well resolved.

MMR status has relationship with the expression of CD4, CD8 and MHC class I molecule, however, the potential mechanism remains unclear. There might be correlations between them in transcriptional or post transcriptional levels, nevertheless lack of evidence. KRAS mutation and BRAF mutation are two important mutations that affect immunological and clinical outcomes of CRC patients. BRAF V600E mutations are overrepresented in dMMR tumors and can be used to rule out Lynch syndrome, while KRAS mutations (in codons 12 or 13) are inversely correlated with dMMR status [32, 33]. Those pMMR CRC patients exhibiting a KRAS or BRAF mutation had the poorest prognosis [34]. The above two aspects were not involved in our study, which are defects of this research and need further investigations.

Previous studies have shown that CRC patients with dMMR status, higher MHC class I expression, and higher CD8^+^ T cell and NK cell infiltration had a better prognosis [35–38]. Our results suggested a loss of MHC class I expression in dMMR group, revealing the complexity of immune related prognostic factors. Multivariate analysis should be carried out to determine the independent prognostic factors. However, due to the limited samples and the fact that most of the patients were in early stage without relapse or death, we failed to make the corresponding research, which should be implemented in the future.

Now, MMR status is thought to be a gold standard for prediction of clinical benefit from immune checkpoint blockade in CRC patients [4]. However, some other predictors should also be explored to improve the predictive efficiency. Tumor-infiltrating lymphocyte (TIL) and PD-L1 have been explored, whereas were not significantly associated with overall survival (OS) or progression-free survival (PFS) [4]. On the basis of our data, MHC class I molecules as another predictor in combination with MMR status is strongly recommend, that’s to say dMMR status with high MHC class I expression might be the most appropriate candidate for immune checkpoint inhibition therapy.

## Conclusions

Taken together, based on our study, high CD8 and low MHC class I expression suggests the contradiction and complexity of immune microenvironment in dMMR CRC patients. Some other immunocytes such as CD56^+^ cells might also participate in the process of immune checkpoint blockade, whereas need further investigations. Besides, dMMR status with high MHC class I expression might be the best candidate for immune checkpoint inhibition therapy.

## Abbreviations

CRC: colorectal cancer
dMMR: mismatch repair-deficient
pMMR: mismatch repair-proficient
MSI: microsatellite instability
MSS: microsatellite stable
PD-1: programmed death-1
PD-L1: programmed death ligand-1
CTLA-4: CTL-associated antigen-4
CTL: cytotoxic lymphocyte
TIL: tumor-infiltrating lymphocyte
OS: overall survival
PFS: progression-free survival
